# Effects of cervical muscle fatigue on the perception of the subjective vertical and horizontal

**DOI:** 10.1186/2193-1801-3-78

**Published:** 2014-02-08

**Authors:** Guy Gosselin, Michael J Fagan

**Affiliations:** School of Engineering, University of Hull, Cottingham Road, Kingston-upon-Hull, HU6 7RX UK

**Keywords:** Muscle fatigue, Field-dependency, Rod and frame test

## Abstract

**Introduction:**

Cervical functional capacity outcome measures that are simple and reliable are urgently needed in order permit accurate assessment/reassessment during treatments and rehabilitation. Induced neck muscle fatigue has been shown to alter functional capacities such as balance and kinaesthetic sense in the standing posture. The Rod and Frame Test has also shown promise as a method of assessing the effects of chronic neck pain and injury, but currently only in the sitting position. The objectives of this project were therefore 1) to validate the computerised rod and frame test in the standing posture, and 2) to measure the effects that different cervical muscle fatigue protocol would have on the assessment of the subjective visual vertical and horizontal.

**Method:**

The validation of the standing computerised rod and frame test in the standing posture was obtained by comparing results (n = 74) between the sitting and standing positions with the Spearman’s correlation coefficient. In addition, agreement between the two methods was analysed with the Bland-Altman method.

Participants (n = 56) resisted with their neck muscles approximately 35% maximum isometric voluntary contraction force for 15 minutes on a purpose built apparatus in eight different directions. Wilcoxon signed rank tests analysed changes in horizontal and vertical rod and frame test between the neutral and all different directions of contraction. The changes of recorded unsigned vertical and horizontal errors for the combined frame condition in all situations of isometric contraction were analysed with two respective one-way repeated measures analysis of variance (ANOVA).

**Discussion:**

The Spearman’s rho and Bland-Altman plots show that the Rod and Frame Test works equally well in sitting and standing positions.

After muscle contraction, there were significant increases in error in all participants for both horizontal and vertical rod and frame tests, except after flexion. These errors were predominantly present after fatigue of muscles in the coronal plane of contraction. Proprioception alone cannot explain the difference in the rod and frame results between different muscle groups. It is suggested that an evolutionary advantage of developing improved subjective verticality awareness in the same direction as the main visual field could explain these findings.

## Introduction

There is a growing body of evidence indicating that after neck injury some parameters associated with cervical functional capacities, such as altered eye movement control, kinaesthetic sensibility, or other problems associated with distorted postural control, the change in altered balance or increased errors in the perception of the visual vertical and horizontal, may not return to the pre-injury state (Roijezon et al., 2010; Yu et al., 2011; Treleaven, 2008). In such situations, few clinicians have access to the sophisticated equipment necessary to accurately assess the neck’s functional capacity. New, simpler and more accessible assessment protocols are urgently needed (Humphreys, 2008).

To that effect, some researchers have developed laboratory protocols that fatigue neck muscles in an attempt to reproduce impairments observed in subjects that have suffered a neck injury (Duclos et al., 2004; Gosselin et al., 2004; Schieppati et al., 2003; Stapley et al., 2006) or experienced cervical mechanical stress (Field et al., 2008; Gosselin and Blouin, 2000). Many of these protocols involve the subjects being tested in the standing position.

Recently, the Rod and Frame Test (RFT) or tests measuring the perception of the subjective visual vertical (SVV) and subjective visual horizontal (SVH) have been used to study the functional effects of either neck pain or whiplash (Bagust et al., 2005; Grod and Diakow, 2002; Docherty et al., 2012). The RFT is a measure of perceptual style, and advances in technology have permitted the development of a modern version of the classic sitting rod and frame test by using a computer and video goggles described as the Computerised Road and Frame Test (C-RFT) (Bagust, 2005). Eventually, Docherty and Bagust improved the C-RFT with the use of two dots instead of a rod-line (C-RFT^dot^) thereby decreasing the visual cues due to screen pixilation seen on the rod during the C-RFT (Docherty and Bagust, 2010).

The first goal of this work was to confirm that a modified C-RFT^dot^ method could be used reliably while participants were standing. The sitting C-RFT^dot^ test methodology as developed by Bagust (2005) was modified in order to 1) decrease the possible proprioceptive cues provided by the sitting position and by both arms touching the computer keyboard and mouse, and 2) reproduce posturographic protocols that are used in many laboratories enabling the experimenters to combine two or more tests in the standing posture. The second goal was to investigate if fatiguing different neck muscle would alter healthy subject’s ability to perceive the subjective visual vertical and horizontal.

## Method

### Validation of C-RFT^dot^ in the standing posture

#### Design and subjects

**Figure 1:**
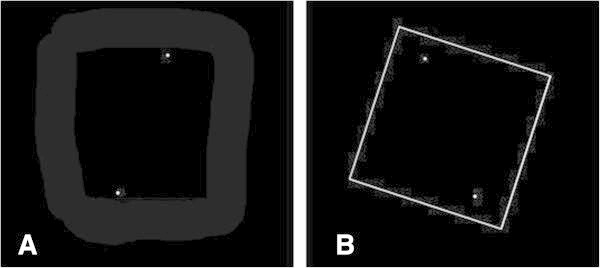
**Computer sceen capture of test. A** Dots. **B** Dots and tilted frame (at +18°).

Seventy (n = 74) healthy male volunteers (23 ± 2 years old; weight = 89.2 ± 6.2 kg, height = 1.82 ± 3.5 m) were recruited from local amateur rugby league and football teams. None were compensated for participating in the study. Inclusion criteria included absence of injuries within the last six months, no visual disturbance, not wearing spectacles and being fit to play. Ethical approval was obtained from the University’s Ethics Committee and all participants signed a consent form after reading an information sheet. Participants were randomised into two equal groups (group A: n = 37; group B: n = 37) according to their surname. All subjects took the C-RFT^dot^ both sitting (T_1_) and after 15 minutes rest in the standing position (T_2_). The order of the tests was determined by a list randomiser (http://www.random.org). There was no time constraint to perform the tests. Subjects performed all tests in a darkened room wearing computerised video goggles (Shenzen EOS Electronics Co, Hong Kong) which effectively simulated the viewing of a 183 cm screen from a 2 m distance. Eye patches attached to the video goggles were used to help limit peripheral vision. Participants looking straight at a dark computer screen were presented with a white square acting as the frame while the head was held in the Frankfort plane (Olivier and Du Toit, 2008) (Figure 1).

#### C-RFT^dot^ method

**Figure 2:**
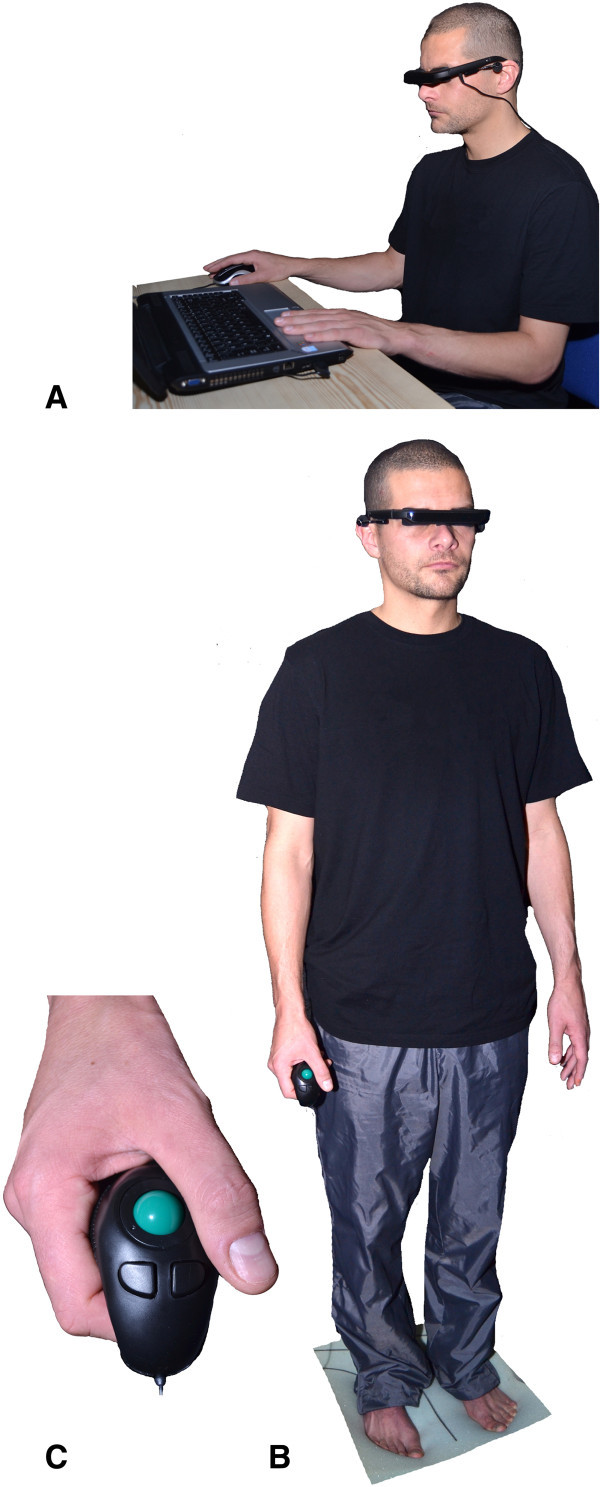
**C-RFT** ^**dot**^ **positions. A**. Participant is sitting at a desk and controls the mouse with the right hand while the left hand validates the trial. **B**. Standing position. The participant controls the dots and validates the trial with one hand. **C**. Close up view of the hand held mouse in the participant’s hand.

Inside the square two superimposed dots were shown. The subjects were instructed to use the right and left computer mouse buttons in order to move the dots around their imaginary centre in clockwise or counter clockwise paths. The dots’ movements were made in 0.5° increments up to a maximum of 30° rotation from the vertical in either direction. A pre-programmed session of 18 situations was presented where there was 1) an absence of the square; 2) the square frame levelled (0°, frame°); 3) square frame angled clockwise +18° (frame^+18^); or 4) square frame angled counter clockwise -18° (frame^-18^). All situations recurred randomly four times as determined by the programme and two additional practice situations completed the test with the dots angled clockwise (+20°) or counter clockwise (-20°) (Docherty and Bagust, 2010). Participants were asked to move the dots as close as possible to their perception of the gravitational vertical or horizontal. Once they had confirmed the alignment using the space bar, the image would clear and the next sequence would appear. Participants would take on average between two and four minutes to complete the test. The second set (T_2_) of experiments was performed during quiet stance with the arms relaxed to the side of the body. Participants held a hand held mouse to control the dots and validate the test sequence which permitted the arms to remain at the side of the body (Figure 2).

### Neck muscle fatigue protocol

**Figure 3:**
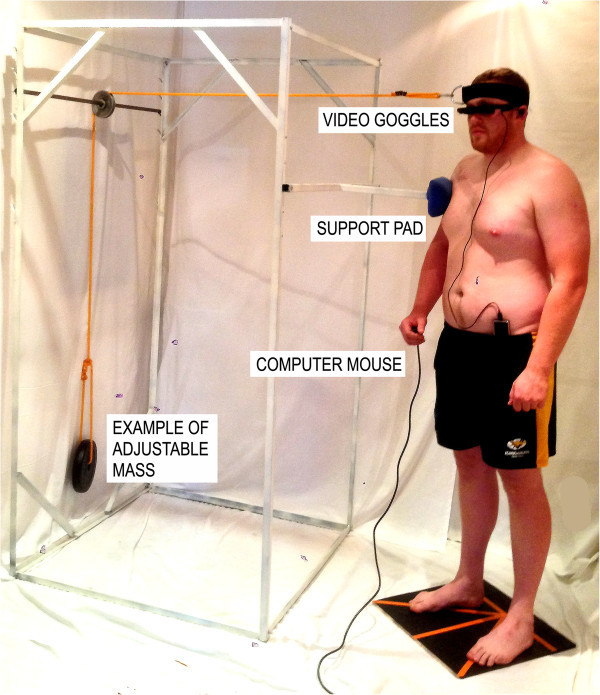
**Experimental setup showing the participant performing an isometric contraction.** The orientation of contraction is in the left posterior oblique direction (LPO). The participant is holding a computer mouse in the right hand. The effort is produced against a cable placed over a pulley to an adjustable mass.

**Figure 4:**
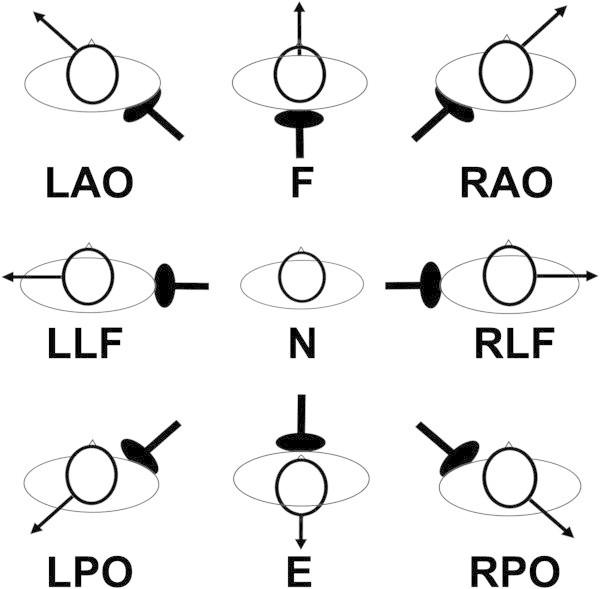
**The orientation of efforts.** Neutral position (N). Extension (E), right posterior oblique (RPO), right lateral flexion (RLF), right anterior oblique (RAO), left posterior oblique (LPO), left lateral flexion (LLF), left anterior oblique (LAO) and flexion (F).

Fifty six (n = 56) participants that took part in the validation study volunteered to participate on different days in the neck fatigue experiment. None were compensated for participating in the study. Participants were randomised into two equal groups (group A: n = 28; group B: n = 28) according to their surname. Neck muscle fatigue was induced by the participants undertaking isometric contractions (Schieppati et al., 2003; Gosselin et al., 2004). They were asked to stand comfortably, the feet comfortably apart, with their arms to their side and leaning slightly towards a support pad. This was provided to help stabilise body movement during the experiment, and was part of a custom built supporting structure (Figure 3). A head weight training harness was placed on the participant’s head, from which a cable extended horizontally and was attached via a pulley system to an appropriate mass. No significant contraction of the body thoracic or lumbar muscle chain below the level of the support was assumed required. A marker was attached to the pulley and the experimenter observed if the pulley remained co-planar with a reference point fixed to the supporting structure. During the isometric contraction, the experimenter would give a verbal cue in order for the participant to either increase or decrease the cervical muscle force against the weight thereby maintaining a static head/neck position. Eight different positions were used each at a 45° offset from the previous one (Figure 4). The orientation of efforts were in extension (E), right posterior oblique (RPO), right lateral flexion (RLF), right anterior oblique (RAO), left posterior oblique (LPO), left lateral flexion (LLF), left anterior oblique (LAO) and flexion (F) (Figure 4). In order to decrease bias due to a participant’s overexertion, the experiment was conducted over two days. On day one, Group A was tested in four different directions each at 90° from the other: E, F, RLF, LLF; on day 2: RPO, LPO, LAO, RAO. The order was reversed for Group B.

The load set on the cable was approximated to 35% of the maximum isometric voluntary contraction as used by Stapley and Schieppati (Stapley et al., 2006; Schieppati et al., 2003) (Table 1). In order to avoid risks of injuries during maximum voluntary contraction measurements, the load was instead calculated individually for each participant by using the isokinetic neck strength profile of elite rugby players and healthy adults (Olivier and Du Toit, 2008; Hogrel et al., 2007). The mass in kilograms required for each participant for a particular movement was then obtained by dividing the peak torque presented in the database by the participant’s neck length and gravitational constant (9.81 m/s^2^). Neck length was measured from the spinous process of the vertebral prominence (C7) to the occipital notch at the base of the skull, while the head was held in the Frankfort plane (Olivier and Du Toit, 2008).

**Table 1:** **The masses and loads used by the participants for a particular movement**

Direction	E	RPO	RLF	RAO	LPO	LLF	LAO	F
**Average torque (Nm)**	56.2	59.5	59.7	50.5	59.5	58.9	50.5	38.9
**Peak mass (kg)**	54	57.2	57.4	48.6	57.2	56.6	48.6	37.4
**35% Peak mass (kg)**	18.9	20.0	20.1	17.0	20.0	19.8	17.0	13.0

Due to the absence of normative data for the oblique contractions, we averaged the torque from either the extension and lateral flexion or the lateral flexion and flexion. For example, the RPO load was determined by averaging the E and RLF torques.

### Isometric muscle contraction and C-RFT^dot^ procedures

The participants stood in a relaxed stance in one of the predetermined positions with their body approximately 2 cm away from the padded vertical support of the apparatus. The C-RFT^dot^ was started. Once the C-RFT^dot^ completed, the head weight training strap was applied to the head and the appropriate weight placed at the end of the cable. During the neck extension muscle sequence, the participants were instructed to lean against the padded vertical support and thus maintain the position of the head and neck, and to readjust the position should the experimenter give them a verbal cue.

After 15 minutes of isometric contraction, the experimenter immediately removed the head weight training straps from the participant’s head and asked the participant to hold a comfortable standing position. Once their position had stabilised, the second C-RFT^dot^ test was started. Participants were allowed 15 minutes recuperation between experimental situations. Overall, the experimentation of the four situations lasted between 2.15 to 2.45 hours.

### Analysis

Each participant’s errors in aligning the rod were used to calculate the absolute means or unsigned or signed values for each of the eight test situations. The direction of the error was determined by the average signed results which indicated the direction of errors whilst the magnitude was determined by the absolute errors. All analyses were performed using SPSS 17.0 and GraphPad Prism 6. Data were tested for normality using the Shapiro-Wilk test. The data are given as median ± standard deviation (SD). As our data were not normally distributed, we looked for correlation between sitting and standing C-RFT^dot^ results with the use of the Spearman’s correlation coefficient test. Furthermore, agreement between the two methods was analysed with the Bland-Altman method on signed errors.

Wilcoxon Signed-Rank tests were used to determine if there were differences in the absolute C-RFT^dot^ errors between the neutral positions and all other eight directions of contraction. Wilcoxon signed rank tests therefore analysed changes in horizontal and vertical C-RFT^dot^ errors between the neutral and eight different directions of contraction. The changes of recorded C-RFT^dot^ unsigned (absolute) vertical and horizontal errors for the combined frame condition in all eight situations of isometric contraction were analysed with two respective one-way repeated measures analysis of variance (ANOVA) with Greenhouse-Geisser (1 factor, 8 levels). The Bonferroni correction was used as post-hoc tests. The use of a parametric test for non-normally distributed data can be justifiable because, even with non-normal distributions at the participant level, with a large enough sample size, such as in our case (n = 56), the distributions of the sample means become sufficiently normal for the ANOVA to be robust enough to analyse the data (Lumley et al., 2002). Significance levels were set at 0.05.

## Results

### Validation

**Figure 5:**
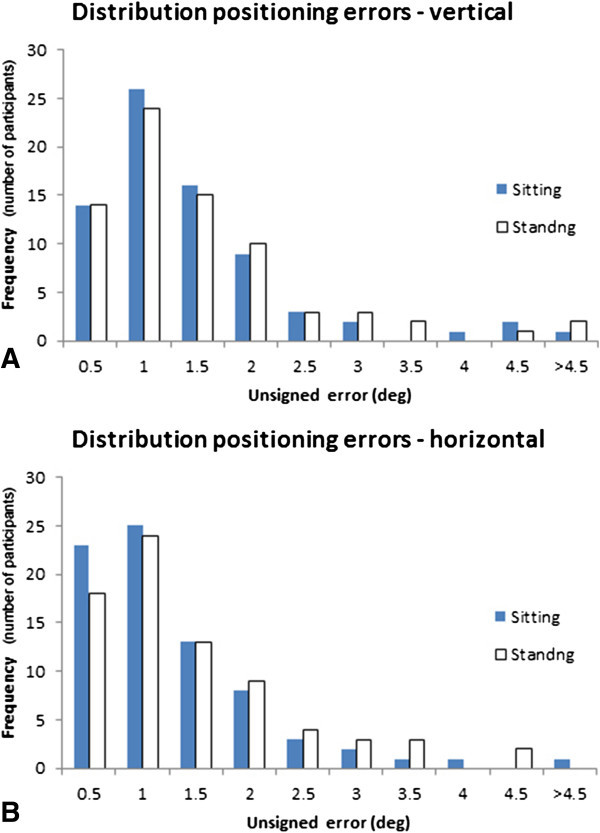
**Distribution of the unsigned errors for the combined frame** ^**+18**^ **and frame** ^**-18**^ **values. (A)** Vertical, **(B)** Horizontal.

**Figure 6:**
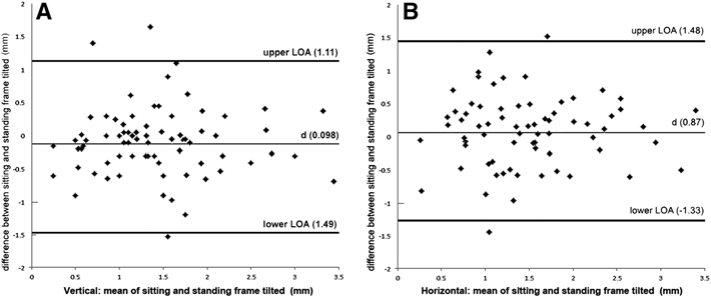
**Bland-Altman analysis of agreement between sitting and standing C-RFTdot results, (A) Vertical mean of sitting and standing frame tilted (mm) showing the 95% limits of agreement between 1.49 mm and 1.11 mm, (B) Horizontal mean of sitting and standing frame tilted (mm) showing the 95% limits of agreement between -1.33 mm and 1.48 mm horizontally.**

Shapiro-Wilk test shows that the recorded positioning errors are not normally distributed (*p* < .001), as shown in Figure 5. The results suggest that the relationship between the C-RFT^dot^ absolute errors in the sitting and standing positions is highly significant for the SVV (rho = 0.982, *p* = 0.01) and for the SVH (rho = 0.950, *p* = 0.01). The Bland-Altman analysis indicates that the 95% limits of agreement between the two methods ranged from -1.49 to 1.11 vertically and -1.33 and 1.48 horizontally (Figure 6). The two methods consistently provide similar measures.

### RFT^dot^ after isometric contraction

Wilcoxon Signed-Rank tests showed there were statistically significant increases in errors in all subjects both for the horizontal and vertical C-RFT^dot^, except after flexion both in the horizontal and vertical C-RFT^dot^ (Tables 2 and 3).

**Table 2:** **Combined horizontal +18** ^**+**^ **and -18 scores for each isometric contraction direction**

Direction	Mean/SD	Median	IQR	CI 95%	z	***p***
**E**	2.22 ± 1.0	2.3	1.2–2.9	1.9–2.4	-4.026	.001
**RPO**	3.8 ± 0.8	3.9	3.3–4.4	3.6–4.0	-6.314	.001
**RLF**	8.22 ± 4.1	7.2	4.8–12.2	7.1–9.3	-6.535	.001
**RAO**	4.35 ± 2.5	3.8	2.8–4.8	3.7–4.9	-6.313	.001
**LPO**	4.32 ± 2.4	3.9	2.4–6.2	3.7–4.9	-6.488	.001
**LLF**	7.67 ± 3.6	7.1	4.8–9.5	6.7–8.6	-6.568	.001
**LAO**	3.78 ± 1.8	2.9	2.4–5.8	3.3–4.2	-6.163	.001
**F**	1.76 ± 0.8	1.8	1.4–2.2	1.5–1.9	-.341	**.733**

Table shows mean and standard deviation, median, Interquartile range (IQR), lower and upper 95% confidence interval of the difference, and the Wilcoxon paired rank z statistic and significance.

**Table 3:** **Combined vertical +18 and -18 scores for each isometric contraction direction**

Direction	Mean/SD	Median	IQR	CI 95%	z	***p***
**E**	2.29 ± 1.0	2.2	1.6–3.0	2.2–2.6	-2.015	.044
**RPO**	3.86 ± 1.1	3.8	3.4–4.2	3.5–4.1	-6.520	.001
**RLF**	6.82 ± 2.6	7.1	4.2–9.4	6.2–7.6	-6.567	.001
**RAO**	4.27 ± 2.7	3.5	2.2–4.8	3.6–5.0	-6.309	.001
**LPO**	4.5 ± 1.63	4.4	3.8–4.8	4.1–4.9	-5.618	.001
**LLF**	7.01 ± 3.0	6.2	4.3–9.5	6.2–7.9	-6.567	.001
**LAO**	3.82 ± 1.3	3.8	3.4–4.2	3.5–4.2	-5.939	.001
**F**	1.57 ± 0.75	1.5	1.3–1.66	1.4–1.8	-.029	**.977**

Table shows mean and standard deviation, median, Interquartile range (IQR), lower and upper 95% confidence interval of the difference, and the Wilcoxon paired rank z statistic and significance.

**Figure 7:**
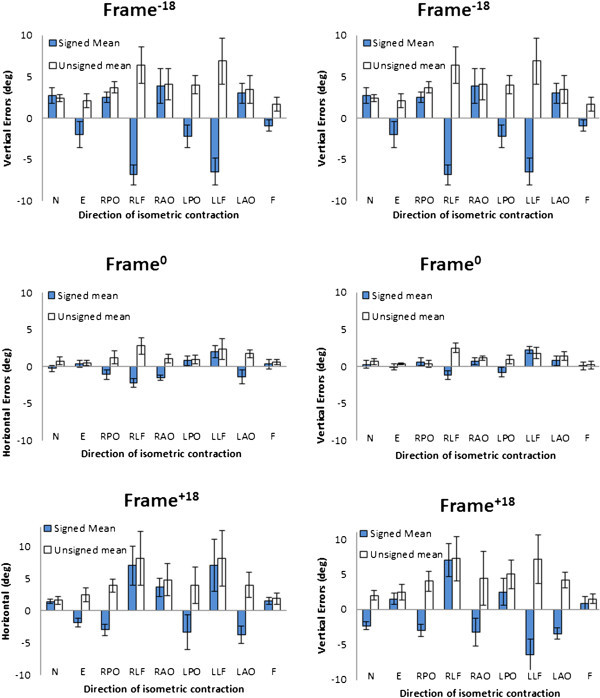
**Vertical and horizontal errors with the frame at frame** ^**-18**^ **frame** ^**0**^ **and frame** ^**+18**^ **.** Errors presented in degrees with standard deviations.

Vertical and horizontal errors with and without tilted frames are shown in Figure 7. When no frame was present, there were significant differences (*p* < .01) in the C-RFT^dot^ after contractions in all directions except after E and F on the horizontal test and E, F, RPO and LPO on the vertical test. The effect of tilting the frame 18° clockwise or counter clockwise caused significant increased positioning errors in all situations (*p* < .001) although there was no difference between the unsigned means of the clockwise and counter clockwise tilted frame for both horizontal and vertical results. More importantly, the direction of contraction did not influence the sign of the positioning errors.

**Figure 8:**
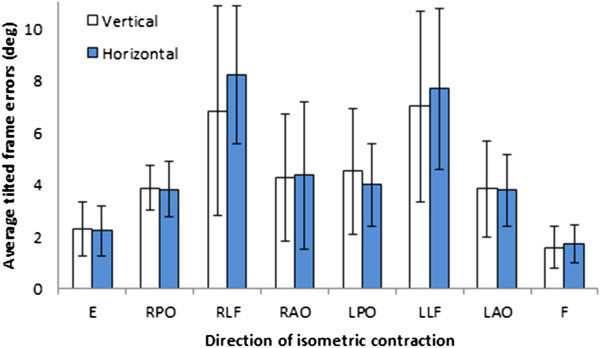
**The vertical and horizontal unsigned combined +18 and -18 scores for each isometric contraction direction.** The largest positioning errors in mm are seen after lateral contraction. Error bars are the standard deviation.

**Figure 9:**
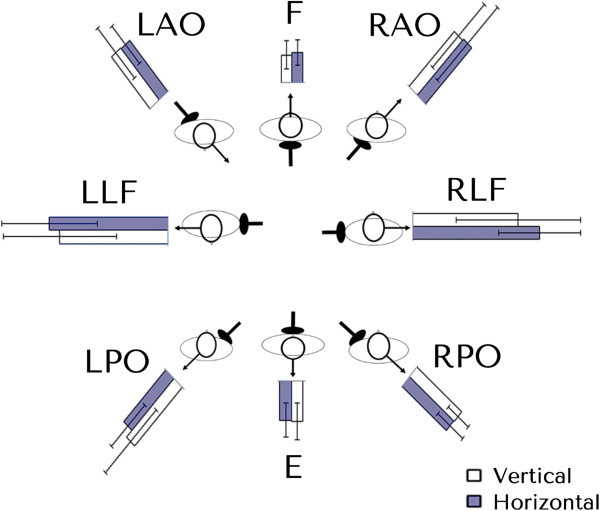
**Graphical representation of the vertical and horizontal combined +18 and -18 scores for each isometric contraction direction.** Extension (E), right posterior oblique (RPO), right lateral flexion (RLF), right anterior oblique (RAO), left posterior oblique (LPO), left lateral flexion (LLF), left anterior oblique (LAO), and flexion (F).

When frames were present the one-way repeated measures ANOVAs with Greenhouse-Geisser corrections determined that different directions of 15 minutes isometric contractions produced significant changes between differences in C-RFT^dot^ (vertical errors: *F*(4.176, 233.830) = 55.272, *p* < .001; horizontal errors: *F*(3.839, 215.005) = 50.699, *p* < .001). Post hoc tests using the Bonferroni correction revealed that changes in C-RFT^dot^ errors were predominantly present in the transverse plane of contraction for both the C-RFT^dot^ vertical and horizontal (Figure 8). There were no significant differences between C-RFT^dot^ horizontal E and F contractions in the sagittal plane (*p* > 0.6) nor were there C-RFT^dot^ vertical mean differences between the RPO, RAO, LPO; RLF, LLF; LPO, RPO, LAO. Figure 9 helps to visualise the differences vertical and horizontal combined unsigned errors.

## Discussion

Our results showed that participants obtained highly correlated scores on the C-RFT^dot^ whilst sitting or standing, but this nonetheless did not confirm agreement between the two methods. The Bland-Altman test was designed specifically to test such an agreement between two experimental methods (Bland and Altman, 1986; Bland and Altman, 2010). We therefore considered both sitting and standing situations as field methods and plotted differences against their mean value. (Since we were comparing different test positions, it was not considered appropriate to assign the sitting position as the reference (Gold Standard) and plot differences against that (Mantha et al., 2000)).

Bagust (Bagust et al., 2005) found no significant difference between the no frame and frame^0^ whilst testing participants in the sitting position. Our results indicate that even without a frame or in the presence of a frame^0^, a neck fatigue protocol does indeed affect the participant’s ability to perceive accurately the subjective vertical and horizontal in all directions of contraction, except after contractions in the sagittal plane (extensor and flexion). Panichaporn also reported this absence of significant change after fatiguing neck extensor muscles at 33.3% MVIC for five minutes (Panichaporn et al., 2013). It is interesting to note that although neck extensor and flexor muscles fatigue did not change the C-RFT^dot^ results, the muscles themselves are quite different from each other both in structure and function. The density of muscle spindles is higher in the small intrinsic, deep dorsal and suboccipital cervical muscles than in other cervical muscle groups which accounts for their important role in proprioception (Peck et al., 1984; Rix and Bagust, 2001). These findings suggest three main effects. Firstly, proprioception alone cannot explain the difference in C-RFT^dot^ scores between muscles fatigued in the sagittal plane and muscles fatigued in the frontal or oblique planes. This is supported by Funabashi’s findings that the use of a neck brace does not provide sufficient afferent input to change a healthy subject’s perception of visual verticality (Funabashi et al., 2011). Additional support for the visual inputs overriding cervical proprioception has been demonstrated in various studies (Karnath et al., 2002; Golomer et al., 2005). For example, Schieppatti showed that the effect of the neck muscle fatiguing contractions did not significantly affect postural sway when vision was allowed (Schieppati et al., 2003). Secondly, these results also support the rejection of the standard model of peripheral fatigue as inadequate to explain the selective effects on the C-RFT^dot^ scores (Noakes et al., 2005).

Lastly, studies on primates has shown that in simpler forms, the midbrain constitutes a mechanism capable of organizing general orienting movements of eyes, head and trunk within the visual fields and controlling associated patterns of contraction in the proximal musculature. The improved perception of vertical has been reviewed in discussion of chimpanzees and hominids bipedalism where this developed mechanism in demanding positions has been suggested to rely on an innate spatial gravitational self-awareness in relation to the ground reaction force (Stanford, 2006; Skoyles, 2006). Presumably the arboreal habitat of early primates imposed selective pressure which favoured the evolution of vision resolving special problems during locomotion. For specific motor behaviour such as fixating a target, it is essential to identify accurately the object in relation to the head. Primates’ brain use abstract, neural imaging of space between sensory and between motor output. These representations seem to be arranged in non-retinal, egocentric coordinates. Therefore, these egocentric references were shown for some time to be intimately associated with perception of body orientation in the sagittal plane such as in the subjective straight ahead experimentations (Karnath et al., 2003; Karnath, 1994). We therefore suggest that the absence of effects after contractions of muscles in the sagittal plane may be related to an evolutionary advantage of developing improved subjective verticality awareness in the same direction as the main visual field even in the presence of disturbances such as muscle fatigue or injury. It has also been reported since the early twentieth century that maximum acuity occurred with horizontal and vertical orientation (Emsley, 1925). Latto suggested that the dominance of visual orientation in perception was that more Hubel and Wiesel orientation detectors in the visual cortex were turned to horizontal and vertical than to oblique lines and edges (Atto and Russel-Duff, 2002). In addition, even though 15 minutes of muscle contraction did increase the errors recorded in the plane of contraction when a frame was present, contrary to other studies, there was no significant difference in the direction of errors recorded (Docherty et al., 2012; Docherty and Bagust, 2010). This phenomenon is reminiscent of motor post-effects seen after short duration isometric contractions (Duclos et al., 2004). The neurophysiological processes underlying these observations are unknown but are similar to observed displacement along the same plane seen in the Kohnstamm phenomenon (Ivanenko et al., 2006).

Takasaki recently studied the minimum repetitions for stable measures of visual dependency using the C-RFT^dot^ (Takasaki et al., 2012). He concluded that instead of the usual four repetitions, five should be used so that the C-RFT^dot^ could give consistent measures of deviation from the vertical in asymptomatic healthy individuals. However, during the validation part of our project, we consistently obtained comparable results to previous C-RFT^dot^ reports which confirms that our method was acceptable (Docherty et al., 2012; Docherty and Bagust, 2010). The higher minimum numbers of repetitions reported by Takasaki could be due to the additional visual feedback provided to their participants by the modified computer program they used in their experiment. Instead of presenting participants with just a white tilted/untilted square frame and two dots on a black background as we have done in our own experiment, Takasaki added a control panel interface on the screen with which the participants were required to move the dots by dragging and turning a button created on the lower right screen using a computer mouse. This additional object in the visual field represents a significant difference in the methods used because the previous C-RFT^dot^ studies have attempted to minimise participant’s visual cues whilst Takasaki actually increased visual cues. We therefore feel confident that our results are a true representation of the participant’s capacities.

It has been shown that high-level athletes participating in open-skills activities specifically involving contact were more field-dependant compared to medium level athletes (Liu, 2003; Liu, 1996). Our participants did not appear to be particularly field-dependant although their results showed less variance than previous C-RFT^dot^ studies. This could be attributed to the highly homogenous group of participants playing mostly the same sport with similar age, skills and level of fitness compared to previous studies presenting more heterogeneous volunteers. A more heterogeneous group of participants or a more field-dependent group of participants could have produced different results. The participants in this study were men therefore caveats should be stated before our results can be applied to other population groups such as neck pain sufferers because woman are more likely to suffer from neck pain than men (Cote et al., 2004).

Our results show that in our participants different muscle groups react differently to disturbances such as isometric muscle contraction. We already know that balance is altered after neck extensor muscle fatigue protocols (Schieppati et al., 2003; Duclos et al., 2009; Gosselin et al., 2004) or after whiplash injuries (Stapley et al., 2006; Field et al., 2008). What is still unknown is what effect injuries to specific muscles groups will have on these functional properties. We have recently completed a study with elite amateur rugby league players on the effects of cervical muscle fatigue protocol on balance (Gosselin et al., 2014). The results show a clear trend from the highest velocity after posterior muscle groups were contracted (E, ROA, RPO) towards the lowest velocity change after flexor muscles contraction (F, LAO, RAO). These results are quite striking when placed in context with the present study as we observe for the first time that neck flexor and extensor muscle groups do not appear to play a significant role in our space awareness abilities as initially thought which support the use of the C-RFT^dot^. We have shown that extensors and lateral flexors appear to be major contributing factors to cervical functional capacities These findings represent important new elements that should be investigated further in order to develop clear outcome and rehabilitation protocols.

## Conclusion

Our experiment has demonstrated that both sitting and standing C-RFT^dot^ methods produce statistically identical results. Furthermore, 15 minutes of constant neck muscle contraction at approximately 35% MVIC in eight different directions in the standing posture increased significantly the C-RFT^dot^ positioning errors in all directions except in the sagittal plane. Proprioception alone cannot explain this phenomenon and it is suggested that an evolutionary advantage of developing improved subjective verticality awareness in the same direction as the main visual field could explain these findings. Further study on the functional role of muscles acting in the frontal plane is encouraged.
